# Spotlight on Antimicrobial Metabolites from the Marine Bacteria *Pseudoalteromonas*: Chemodiversity and Ecological Significance

**DOI:** 10.3390/md14070129

**Published:** 2016-07-08

**Authors:** Clément Offret, Florie Desriac, Patrick Le Chevalier, Jérôme Mounier, Camille Jégou, Yannick Fleury

**Affiliations:** Laboratoire Universitaire de Biodiversité et d’Ecologie Microbienne LUBEM EA3882, Université de Brest, Technopole Brest-Iroise, 29280 Plouzané, France; clement.offret@univ-brest.fr (C.O.); florie.desriac@polytech-lille.fr (F.D.); patrick.lechevalier@univ-brest.fr (P.L.C.); jerome.mounier@univ-brest.fr (J.M.); camille.jegou@univ-brest.fr (C.J.)

**Keywords:** *Pseudoalteromonas*, antimicrobial metabolites, alkaloid, polyketide, non ribosomal peptide, genome mining, marine host-associated microbiota, probiotic

## Abstract

This review is dedicated to the antimicrobial metabolite-producing *Pseudoalteromonas* strains. The genus *Pseudoalteromonas* hosts 41 species, among which 16 are antimicrobial metabolite producers. To date, a total of 69 antimicrobial compounds belonging to 18 different families have been documented. They are classified into alkaloids, polyketides, and peptides. Finally as *Pseudoalteromonas* strains are frequently associated with macroorganisms, we can discuss the ecological significance of antimicrobial *Pseudoalteromonas* as part of the resident microbiota.

## 1. Introduction

Last October, we celebrated the 20th anniversary of the genus *Pseudoalteromonas* having been split from *Alteromonas* [1]. The genus *Pseudoalteromonas* includes Gram-negative, heterotrophic, and aerobic bacteria with a polar flagellum and has a GC content comprised between 38% and 50% [2]. It belongs to the order Alteromonadales in the γ-Proteobacteria class. The *Pseudoalteromonas* strains require a seawater base for growth and are therefore true marine bacteria. Nowadays, 41 species are assigned to this genus and over 3772 *Pseudoalteromonas* strains are currently listed in the NCBI taxonomy browser. Allowing for rare exceptions, *Pseudoalteromonas* strains are associated with healthy animals or algae. To date, few strains are known as pathogenic or opportunistic. *Flavobacterium piscicida*, reclassified as *P. piscicida* by Gauthier et al., is involved in flavobacteriosis in farm fish [3], while *P. agarivorans* NW4327 was recently reported as pathogenic for the sponge *Rhopaloeides odorabile* [4].

This bacterial genus is of great interest to the scientific community because of (i) its prolific metabolite-producing capacity and (ii) its usual association with macroorganisms [5], leading to a suspected and sometimes documented ecological significance. These two properties may, in fact, be interconnected. According to the hologenome theory [6,7,8,9,10], the holobiont is composed of the host and its associated microbial communities, named microbiota. Such a superorganism gains genetic plasticity and flexibility and therefore appears better equipped to face and adapt to environmental variations. The microbiota is supposed to play a critical role in holobiont homeostasy through its metabolic activities. Moreover, the microbial shielding of the microbiota may defend the holobiont from pathogen settlement and therefore participate in the host protection. The abundance of antimicrobial metabolite described from *Pseudoalteromonas* strains points them out as a key partner in marine invertebrate holobionts.

This review focuses on the antimicrobial compounds produced by *Pseudoalteromonas* strains. It presents a chemical inventory of the *Pseudoalteromonas* antimicrobial metabolites known to date. Their bioactivity will not be discussed because of the lack of a standardized panel of target microorganisms. Indeed, although significant efforts have been made regarding testing procedures, the use of various microbial species and strains is not suitable for a rigorous comparison of antimicrobial activity and potency. Finally, the potential ecological significance of the antimicrobial metabolite-producing *Pseudoalteromonas* is revisited in the light of antimicrobial activity and the origin of the bioactive strains.

## 2. Antimicrobial Metabolites from *Pseudoalteromonas* Species

**Figure 1:**
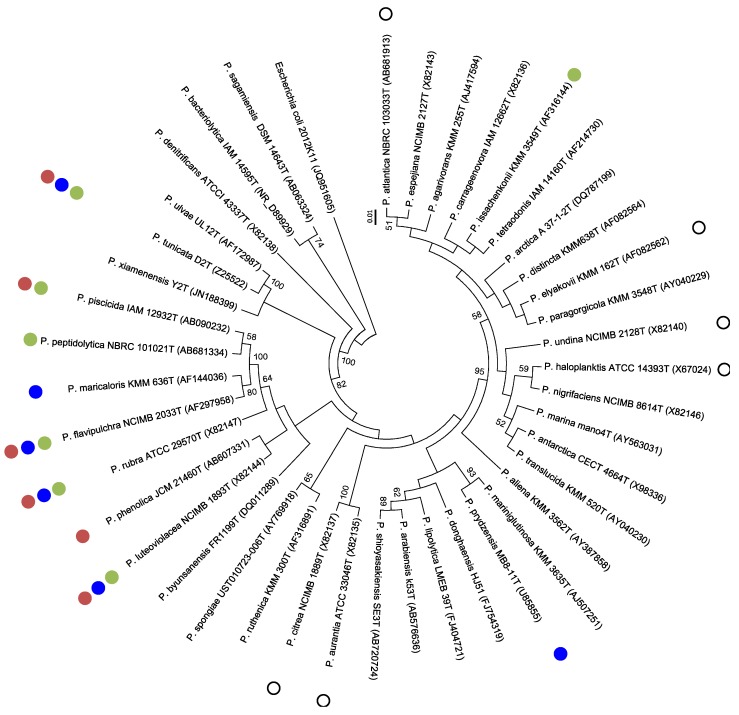
Neighbor-joining tree indicating the phylogenetic relationships inferred from partial 16S rDNA gene sequences (1196 nt) of *Pseudoalteromonas* strains producing either alkaloids (**green**), polyketides (**red**), or peptides (**blue**). Empty circles represent strains producing unidentified antimicrobial compounds. Bootstrap values (expressed as percentage of 1000 replications) > 50% are shown at branching points. The proteobacteria *Escherichia coli* 2012K11 (position 167-1356) was used as outgroup. Bar, 0.01 substitutions per nucleotide.

In marine microorganisms, secondary metabolites are mostly comprised of nitrogenated (56%), acetate- (30%), and isoprene-derived (13%) molecules [11]. On the basis of their biosynthetic pathways, they are classified as alkaloids or peptides, polyketides, and terpenoids [12]. This review will adopt the same classification system for the antimicrobial metabolites produced by *Pseudoalteromonas* strains that way, the antimicrobial metabolites from *Pseudoalteromonas* known to date (*n* = 69) can be categorized as alkaloids (*n* = 32, 46%), polyketides (*n* = 20, 29%), and peptides (*n* = 17, 25%) (Figure 1). The antimicrobial strains that produce these metabolites are affiliated with at least 16 different species. In comparison, only six of the 126 species composing the dominant marine bacterial family, *Vibrionaceae*, are known to produce 29 antimicrobial identified compounds [13].

Among the *Pseudoalteromonas* genus, the metabolite-producing capacity has usually been associated with pigmentation. The antimicrobial metabolite-producing *Pseudoalteromonas* species did not derogate since most of them (>80%) are pigmented and the bioactive metabolites produced have been shown to cause this appearance [14]. For example, *P. tunicata* CCUG 26757 and *P. rubra* DSM 6842 strains produced various pigments involved in their antibacterial and antifungal activity [15,16,17,18,19,20,21]. However, such a correlation is abusive. Indeed pigmentation is the result of the presence of conjugated double bonds in the chemical structure of the antimicrobial metabolite. This structural feature is not a prerogative for antimicrobial activity, as exemplified by bioactive non-ribosomal peptides that are colorless. Hence, this observation should not be laid down as a rule. It is important to keep in mind that non-pigmented *Pseudoalteromonas* strains are also potential bioactive metabolite producer even though it is less frequently observed.

### 2.1. Alkaloids

Alkaloids are biosynthesized from amino acids. They are predominantly made of heterocyclic incorporated basic amine nitrogen. Mostly derived from α-amino acids such as Phe, Tyr, Trp, Lys, and Orn, the biosynthesis pathways of alkaloids remain poorly understood [22]. Alkaloids represent the majority of the antimicrobial metabolites from *Pseudolteromonas*, having been isolated from a vast number of strains belonging to seven species (Figure 1). The antimicrobial alkaloid metabolites from *Pseudoalteromonas* are divided into non-halogenated (Table 1) and halogenated compounds (Table 2).

A number of halogenated alkaloids is produced by marine animals, bacteria, and fungi [23]. Halogenation has now been observed in many natural product biosynthetic pathways and can play a significant role in establishing the bioactivity of a compound [24,25]. Only brominated and chlorinated residues are incorporated into antimicrobial halogenated alkaloids produced by *Pseudoalteromonas* species. They are associated with pyrrole or benzene ring(s) or aliphatic side chain. The bromoperoxidase enzyme catalyzes the oxidation of bromide (Br^−^) to the electrophilic bromonium cation (Br^+^), which in turn attacks hydrocarbons and results in the production of brominated alkaloids [23,26]. Similarly, chloroperoxidases are responsible for the production of chlorinated alkaloids. *P. luteoviolacea* produces four of the 10 brominated alkaloids with antimicrobial activity discovered among *Pseudoalteromonas* strains. Other strains such as *P. peptidolytica*, *P. flavipulchra*, and *P. phenolica* are also described to produce alkaloids with bromine, with *P. peptidolytica* being the only species to produce chlorinated alkaloids.

### 2.2. Polyketides

Polyketides constitute a rich and diverse family of natural compounds from prokaryotes and eukaryotes. By definition, all polyketides are synthesized by condensation of acetyl (also referred to as ketides) or malonyl units via specific enzymes called PolyKetide Synthases (PKS). These enzymes catalyze the condensation of activated acyl derivatives (acyl-CoA) on an existing ketide linked to the enzyme via a thioester bond [56]. Several categories of PKS are described within bacteria, depending on (i) the linear/iterative functioning of the enzyme; and (ii) the way acyl-coA is incorporated (involving or not an Acyl Carrier Protein ACP) [57]. Hence, to date, three PKS types have been recognized. Type I PKS are sequential multifunctional enzymes while type II PKS are iterative and multienzyme complexes. In contrast to type I and II PKS, type III PKS are iterative but do not involve ACP. Until the late 1990s, type III was thought to be plant-specific but recent developments have highlighted that they are also found in microorganisms [58]. PKS have been shown to yield many bioactive molecules, especially antibiotics such as erythromycin [59] and tetracycline [60]. Within the genus *Pseudoalteromonas*, only three different species produce nine groups of polyketides exhibiting antimicrobial activity (Table 3). Indeed, polyketides are at the origin of numerous antimicrobial phenols and, in association with NRPS and Fatty Acid Synthase (FAS) systems, of the well-known thiomarinols produced by *P. luteoviolacea* [61] (Table 3).

### 2.3. Non Ribosomally Synthesized Peptides

Non Ribosomal Peptides Synthetases (NRPS) represent a wide group of multimodular enzymes that polymerize amino acids as well as fatty acids, and α-hydroxy acids [68]. NRPS can also incorporate unusual amino acids (non-proteinogenic) and hence generate a great chemodiversity. Basically, NRPS are divided into modules, each one being responsible for binding one specific amino acid to the peptidic chain. To do so, each module consists of at least three domains. The Adenylation domain (A) activates the amino acid. Then, the Peptidyl Carrier Protein (PCP, also referred to as thiolation domain T) transfers it to the Condensation domain, which incorporates the new amino acid residue into the rest of the peptide via a peptidic bond. For the last module, a final ThioEsterase domain (TE), breaks the NRPS–Peptide bond and releases the product [69]. Furthermore, several tailoring modules can modify the structure of the peptide [70]. As for PKS, the mechanism of synthesis is usually linear but can also be iterative [69,71]. NRPS are frequently associated with PKS and even FAS to generate hybrid molecules, such as the bromoalterochromides produced by several *Pseudoalteromonas* species (Table 4) [72,73]. Actually, many NRPS products are hybrid, meaning these products can be classified as polyketides and non-ribosomal peptides at the same time.

### 2.4. Bacteriocins and Bacteriocin-Like Inhibitory Substances (BLIS)

Bacteriocins and Bacteriocin-Like Inhibitory Substances (BLIS) are proteinaceous antibacterial compounds. They are ribosomally synthesized and exhibit a narrow spectrum of activity, generally limited to closely related strains. In *Pseudoalteromonas*, most of them are large proteins (MW > 100 kDa) (Table 5). Although they fell under this classification, most of them exhibited enzymatic activity ((l)-amino acid oxidase (LAAO)) activity. In many cases, these LAAOs were considered to be a flavin adenine dinucleotide (FAD)-containing homodimeric protein, [76]. LAAOs are widely found in *Pseudoalteromonas* and provide an important ecological function in marine environments [77].

### 2.5. Uncharacterized Chemistry of Antimicrobial Metabolites Produced by Pseudoalteromonas Species

Numerous *Pseudoalteromonas* strains have shown an antimicrobial activity but the bioactive compound(s) remain unidentified [55,85,86,87]. During a global marine research cruise, 15 strains related to *P. ruthenica* have exhibited antibacterial activity against *Vibrio anguillarum* [15]. Three bioactive fractions were detected after ethyl acetate extraction and RP-HPLC splitting. The compounds could not be assigned to any known secondary metabolites produced by pigmented *Pseudoalteromonas* species [15]. Moreover, *P. tunicata* strains inhibiting a variety of common fouling organisms were shown to produce a polar, heat-stable compound [88] and a heat-sensitive 3–10 kDa compound [89]. A cell-free supernatant of a *P. piscicida* strain isolated from a crustacean also showed an antifungal activity [90]. More recently, an antimicrobial anionic protein with an 87 kDa molecular weight was isolated from a culture of *P. piscida* [91]. This hydrophobic compound showed a high content of serine as well as aspartic and glutamic acids, but its amino acid sequence was not defined. Finally, two yellow-pigmented strains of *P. citrea* and *P. aurantia* were shown to exert an antimicrobial activity against bacteria, but their chemical nature has not been elucidated yet [92,93].

### 2.6. Genome Mining Strategies as a Tool to Discover Antibiotics in Pseudoalteromonas

The last two decades were marked by the onset of genomic area with cheaper and faster technologies for genome sequencing. To date, 29,000 complete bacterial genomes have been deciphered and another 31,000 bacterial genome sequences are still in progress under way (GOLD database: http://www.genomesonline.org; date of access: May 2016). This has led to the development of genome-based strategies to discover new drugs and antibiotics [94]. As a consequence, bioinformatic tools have been developed to improve the metabolite-pathway analysis, allowing access to putative metabolites and therefore to the investigation of the biotechnological potential of bacterial strains. Thanks to this genome mining approach, the metabolome can be predicted. Furthermore, bioinformatic tools enable the identification of new and potentially novel compounds via the expression of silent genes. Indeed, it is estimated that less than 10% of secondary metabolite gene clusters are expressed in sufficient amount for detection in current lab conditions [95].

As NRPS, PKS, and hybrid NRPS-PKS pathways exhibit repeated motifs in genomic nuclear sequences, they have led to the development of powerful bioinformatics programs such as antiSMASH [96], NapDos [97], SBSPKS [98], or Np.searcher [99]. Moreover, the description of a specificity-conferring code in the NRPS A-domain [100] has resulted in the development of specialised chemical structure prediction tools, e.g., NRPS predictor2 [101] and NRPS substrate predictor [102]. Another tool dedicated to ribosomally-produced peptides (bacteriocins), BAGEL3 [103] can be useful in a genome mining approach focusing on the discovery of new antimicrobial compounds. The reader is referred to the recent reviews focusing on genomics strategies defined to discover microbial natural products [104,105,106,107]. Combined with this, experimental plans have also been established to characterize potential secondary metabolites according to in silico analyses: isotope labelling, gene knockout, heterologous expression, or transcription activation. For more information on these strategies, the reader is referred to [95,106,108,109].

Despite an increasing number of available *Pseudoalteromonas* genomes and an increased knowledge about their bioactive compounds, only a few studies based on genome mining have been dedicated to the discovery of new antibacterial metabolites [110,111]. A consequent genomic study of 21 antimicrobial marine bacteria, of which seven belonged to the *Pseudoalteromonas* genus, highlighted the potential of pigmented *Pseudoalteromonas* strain as producers of secondary metabolites. Genomic analyses corroborated biochemical results e.g., *Photobacterium halotolerans* S2753 was previously described to produce holomycin [13] and the gene cluster responsible for its biosynthesis was underlined in this study. Concerning *Pseudoalteromonas*, despite a deeper analysis, no potential gene or gene cluster was identified on the bacterial genome of *Pseudoalteromonas ruthenica* [110]. As for biochemical approaches, all efforts made into genome analysis failed to characterize anti-*Vibrio* and *Staphylococcus* compounds in this strain. Added to this, the same team recently applied a coupled metabolomics and genomics workflow to determine the biosynthetic potential of *Pseudoalteromonas luteoviolaceae* [112]. Combined methods allowed rapid identification of new antibiotics and their biosynthetic pathways. Papaleo and co-workers conducted a study on four Antartic strains, two of which belonged to the genus *Pseudoalteromonas*, having inhibitory activity against *Burkholderia cepacia* complex [111]. As antimicrobial activity appeared to be shared and related to microbial volatile organic compounds, they conducted a genomic comparative analysis to point possible common secondary metabolite producer genes. Few candidates (11) were shown to be involved in secondary metabolite biosynthesis, transport, and catabolism, and further analysis had to be performed to clarify the implication of these genes in the antimicrobial activity.

As shown by the small number of reports, genome mining is in its infancy for the genus *Pseudoalteromonas*; however, pioneering studies on this topic highlight the use of the genomics approach in bioprospecting. However, as underlined by these same studies, it should be kept in mind that the reality of these virtual metabolites is to be established. They have to be structurally and functionally characterized. Therefore, the genome mining tools should be considered as tools to orientate metabolite discovery and not as an end in themselves. Nevertheless, further analyses should be carried out to characterize the different pathways and/or the natural products. The recent development of generic tools for *Pseudoalteromonas* genetic manipulation [113], as well as more specific ones [77,114,115,116], will be helpful in this endeavor.

## 3. Ecological Significance in Marine Life

To date, of the 41 *Pseudoalteromonas* species described, 16 (39%) have been shown to produce antimicrobial compounds. When investigating the ecological niche occupied or the isolation origin of these bioactive *Pseudoalteromonas*, we observed that these antimicrobial metabolite-producing *Pseudoalteromonas* were directly associated with macroorganisms except for *P. phenolica*, isolated from seawater (Table 6). Therefore, there is a great temptation to connect *Pseudoalteromonas* antimicrobial properties with its host association. The antimicrobial metabolite producing *Pseudoalteromonas* strains may form a microbial shield and as a result contribute to the protection of their host against pathogens [111].

A plethora of antimicrobial metabolite-producing strains of *Pseudoalteromonas* has been isolated in association with marine invertebrates (Table 6). The most documented hosts belong to *Porifera* (sponges in particular) and *Cnidaria*. The former are known to house great bacterial diversity. *Pseudoalteromonas* strains producing antimicrobial metabolite in vitro have been isolated from animals living in tropical [141], temperate [87,142], and cold seawater [111]. In Cnidarians, the role of *Pseudoalteromonas* in host defense and health has been hypothesized facing the antimicrobial metabolite producing strains of *Pseudoalteromonas* in corals [6,48,143,144]. Such associations with antimicrobial metabolite producing strains of *Pseudoalteromonas* strains are less described in the other *Phyla* except for molluscs (mainly bivalves [84,145,146] and crustaceans [147]).

The suggested role of such bacterial strains has led to the hologenome theory and concept [6,7,8,9,10], in which the holobiont is considered as the true evolutionary unit. Therefore antimicrobial metabolite producing strains of *Pseudoalteromonas* may play a key role in microbiota shaping and microbial shielding of marine invertebrates. Using next-generation sequencing methods, the impact of biotic (pathogen or probiotic) and/or abiotic stresses onto microbiota may be clarified. Such a strategy was recently applied to hemolymph microbiome of oysters [148]. An abiotic stress (temperature) was shown to provoke significant modifications of the microbiome composition while a biotic one (*Vibrio* sp. infection) did not. This microbiome stability has supported the hypothetical role of microbiota in host defense. Exploiting antimicrobial-producing *Pseudoalteromonas* spp. as tools to shape the marine host-associated microbiota along with high-throughput sequencing of host-associated microbiota may elucidate the role of *Pseudoalteromonas* in host defense.

The molecular dialog between antimicrobial-producing *Pseudoalteromonas* and the host immune system is another area that should be investigated. Whether in invertebrates, vertebrates, or algae, macroorganisms have Pattern-Recognition Receptors (PRR) at their disposal to detect and recognize microbial components known as Pathogen-Associated Molecular Patterns (PAMP). In Gram-negative bacteria such as *Pseudoalteromonas*, one of the most potent PAMPs is the LipoPolySaccharide (LPS). Few studies completely defined LPS structures of *Pseudoalteromonas* species [149]. However, it appears that most *Pseudoalteromonas* LPS known to date are composed of a pentaacylated Lipid A instead of a hexaacylated one [149,150,151,152,153,154]. Such a structural difference may provide an advantage to evade PRRs-mediated recognition. Indeed, LPS from various *Pseudoalteromonas* strains were shown to elicit a low immune response and are capable of modulating immune responses in their hosts [150,153].

Finally, in vivo experiments are required to determine the ecological significance of the association between *Pseudoalteromonas* and their source macroorganisms. The major issue is to determine whether antimicrobial-producing *Pseudoalteromonas* provide a real benefit to their host, especially in the context of pathogenic events. In such a case, *Pseudoalteromonas* could stand as a next generation of probiotics for marine aquaculture.

## 4. Conclusions

The genus *Pseudoalteromonas* has a high, if not the highest, proportion of species producing antimicrobial metabolites in the marine bacterial world. However, this bacterial genus is still underexplored at the biotechnological level. Furthermore, the vast majority of *Pseudoalteromonas* spp. have not been found to exhibit pathogenicity. Almost all known antimicrobial-producing *Pseudoalteromonas* spp. originate from healthy marine macroorganisms, suggesting that these strains may participate in the host’s homeostasis. It would be simplistic to link only the associated microbiota and the ensuing microbial shield with the presence of *Pseudoalteromonas* strains. Obviously, the metabolite richness and the genetic plasticity of the symbiont–host relationship result from metabolic and therefore microbial diversity. Nevertheless, the involvement of antimicrobial-producing *Pseudoalteromonas* in microbiota shaping and protection of their host should not be under-estimated, or neglected. In any event, the genus *Pseudoalteromonas* appears as the first or at least one of the leading antimicrobial providers in the marine microbiota. Therefore, *Pseudoalteromonas* strains offer real potential to develop the next generation of marine probiotics and their use as probiotics in aquaculture should be further investigated.

## Figures and Tables

**Table 1:** Non-halogenated compounds from *Pseudoalteromonas* species with antimicrobial activities.

Producers		Products		Sensitive Microorganisms	Ref.
Affiliated Species	Strain	Name	Structure		
*P. peptidolytica*	J010	Korormicin, R: (CH_2_)_7_CH_3_	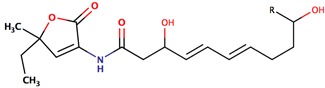	Gram-negative	[27,28,29]
		Korormicin G	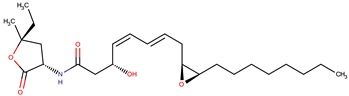		
		Korormicin J R = CH_2_CH_3_	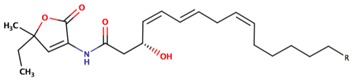		
		Korormicin K R = (CH_2_)_2_CH_3_			
*P. tunicata*	CCUG 267547	Tambjamine	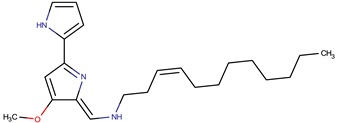	*Staphylococcus aureus, Escherichia coli, Candida albicans, Malassezia furfur*	[20,21,30,31,32,33,34,35]
		Tambjamine YP1	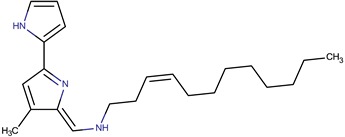		
*P. rubra*	DSM 6842	Prodigiosin	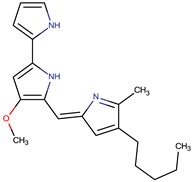	*Staphylococcus aureus, Escherichia coli, Candida albicans*	[15,16,17]
		Cycloprodigiosin	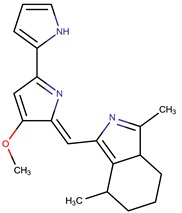		
		2-(*p*-H-benzyl) prodigiosin	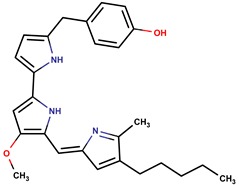		
*P. luteoviolacea*	NCIMB 2035	Violacein R1 = OH, R2 = H	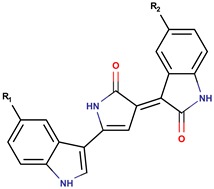	*Staphylococcus aureus, Bacilus subtilis, Bacillus megaterium, Photobacterium* sp., fungi	[15,36,37]
		Oxyviolacein R1 = R2 = OH			
		Deoxyviolacein R1 = R2 = H			
		Indolmycin	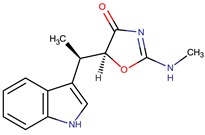	*Staphylococcus aureus*	[15,38]
*P. rubra*, *P. piscicida*	DSM 6842 A1-J11	pentyl-quinolinone R: (CH_2_)_3_CH_3_	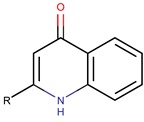	*Staphylococcus aureus, Vibrio anguillarum, Vibrio harveyi, Candida albicans*	[15,39,40,41,42]
		heptyl-quinolinone R: (CH_2_)_5_CH_3_			
		nonyl-quinolinone R: (CH_2_)_7_CH_3_			
*P. piscicida*	NJ6-3-1	Norharman	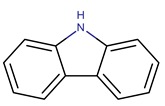	*Bacillus subtilis, Staphylococcus aureus, Agrobacterium tumefaciens, Escherichia coli, Saccharomyces cerevisiae*	[43]
*P. flavipulchra*	JG1	n-hydroxy benzoisoxazolone, 2′-deoxyadenosine	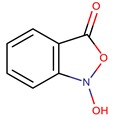	Gram-positive and Gram-negative	[44]
			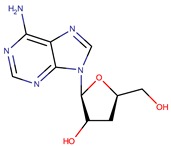		
*P. issachenkonni*	non-referenced	Isatin	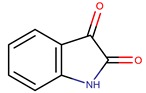	*Candida albicans*	[45,46,47]
*P. piscicida*	OT59	Alteramide A	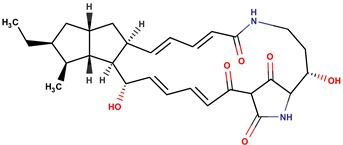	Fungi	[48]

**Table 2:** Halogenated compounds from *Pseudoalteromonas* species with antimicrobial activities.

Producers		Products		Sensitive Microorganisms	Ref.
Affiliated Species	Strain	Name	Structure		
*P. luteoviolacea P. peptidolytica*	I-L-33 J010	Tetrabromopyrrole	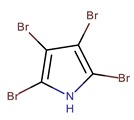	*Staphylococcus aureus, Enterobacter aerogenes, Escherichia coli, Photobacterium fisheri, Photobacterium mandapamensis, Photobacterium phosphoreum, Pseudomonas aeruginosa, Candida albicans*	[27,49]
*P. luteoviolacea*	I-L-33	Hexa-bromo-2,2′-bipyrrole	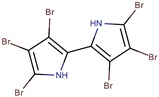	*Photobacterium* sp.	[23,49]
		4′-((3,4,5-tribromo-1*H*-pyrrol-2-yl)methyl) phenol	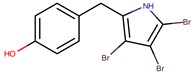	*Staphylococcus aureus*	[27]
*P. luteoviolacea*	2ta16	2,4,6-Tribromophenol	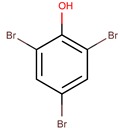	Bacteria and fungi	[50,51]
		2,6-dibromophenol	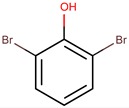	Fungi	[50,52]
*P. luteoviolacea P. phenolica*	I-L-33 D5047	Pentabromopseudilin	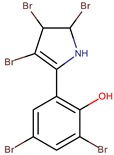	*Staphylococcus aureus Photobacterium phosophoreum*	[23,49,53,54,55]
*P. phenolica*	D5047	2,3,5,7-tetrabromobenzofuro [3,2-*b*]pyrrole	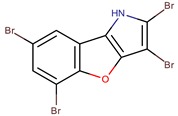	*Staphylococcus aureus, Candida albicans*	[55]
*P. peptidolytica*	J010	Korormicin H, F, I H: R1: (CH_2_)_2_CH_3_ R2: OH R3: Cl F: R1: (CH_2_)_2_CH_3_ R2: Br R3: OH I: R1: (CH2)2CH3 R2: Cl R3: OH	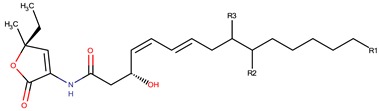	Gram-negative	[27]
*P. flavipulchra*	JG1	6-bromoindolyl-3-acetic acid	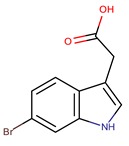	Gram-positive and Gram-negative	[44]

**Table 3:** Polyketides produced by *Pseudoalteromonas* species with antimicrobial activities.

Producers		Products		Sensitive Microorganisms	Ref.
Affiliated Species	Strain	Name	Structure		
*P. phenolica*	O-BC30T	MC21-A	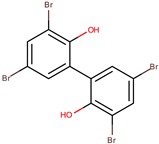	*Staphylococcus aureus, Escherichia coli*	[55,62]
	D5047	4,4′,6-tribromo-2,2′-biphenol	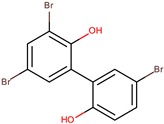		
*P. luteoviolacea*	Non-referenced	2,4-dibromo-6-chlorophenol	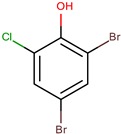	*Staphylococcus aureus*	[63]
	I-L-33	4-hydroxy benzaldehyde (2)	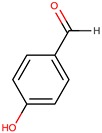	Gram-positive Gram-negative	[49]
		*n*-propyl-3-hydroxybenzoate (3)	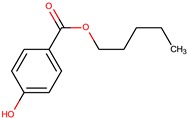		
*P. luteoviolacea*	SANK 73390	Thiomarinols A,C,D,E,F	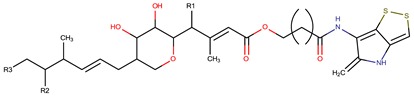	Gram-positive Gram-negative	[61,64,65,66,67]
			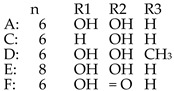		
		Thiomarinol B	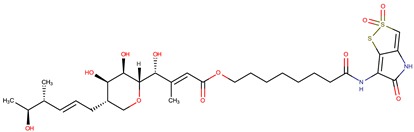		
		Thiomarinol G	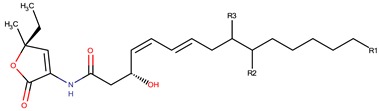		
		Xenorhabdin	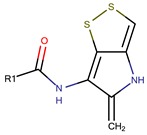		
		R1: decanoyl			
		R1: dodecanoyl			
		R1: *E*-dec-3-enoyl			
		R1: *Z*-dec-4-enoyl			
		R1: *E*-tetradecenoyl			
		R1: *Z*-hexadecenoyl			
*P. flavipulchra*	JG1	*p*-hydroxybenzoic acid	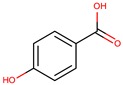	*Bacillus subtilis, Aeromonas hydrophila, Photobacterium damselae, Vibrio anguillarum, Vibrio harveyi*	[44]
		*trans*-cinnamic acid	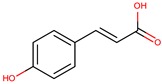		

**Table 4:** Peptidic compounds produced by *Pseudoalteromonas* species with antimicrobial activities.

Producer		Compound		Sensitive Microorganisms	Ref.
Affiliated Species	Strain	Name	Structure		
*P. rubra* *P. flavipulchra* *P. maricaloris*	DSM 6842 NCIMB 2033 KMM 636	Bromoalterochromide A, R = i B, R = i Dibromoalterochromide A, R = ii B, R = ii	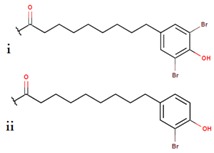	*Bacillus subtilis, Staphylococcus aureus, Enterococcus faecium, Vibrio anguillarum, Candida albicans*	[15,74]
		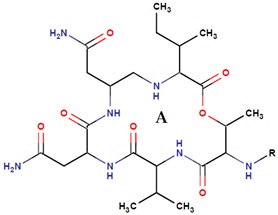		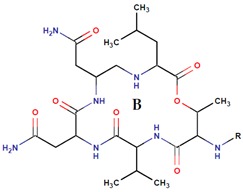	
*P. maricaloris*	KMM 636	cyclo-(isoleucyl-prolyl-leucyl-alanyl)		Bacteria and fungi	[75]

**Table 5:** Bacteriocins and BLIS produced by *Pseudoalteromonas* species.

Producer		Compound			Sensitive Microorganisms	Ref.
Affiliated Species	Strain	Name	Structural Information			
*P. luteoviolacea*	CPMOR-1		l-amino acid oxidase	110 kDa	*Bacillus subtilis, Staphylococcus*	[78]
					*epidermidis, Escherichia coli*	
*P. tunicata*	D2	AlpP	l-Lysine oxidase	190 kDa	Gram positive Gram negative	[79]
*P. flavipulchra*	C2		l-amino acid oxidase	60 kDa	*Staphylococcus aureus*	[80]
	JG1	PfaP	694 amino acids	77 kDa	*Vibrio anguillarum*	[81]
*P. luteoviolacea*	9k-V10			100 kDa	Gram positive Gram negative	[82]
	9k-V9			50 kDa	*Vibrio parahaemolyticus*	[41,42]
*P. prydzensis*	h*Cg*-6		BLIS		Gram negative	
	h*Cg*-42		BLIS		Gram negative	[71,83]
	DIT44, DIT46, DIT9		Amphiphilic compounds	<3 kDa	*Vibrio parahaemolyticus*	[84]

**Table 6:** Marine macroorganisms that host antimicrobial *Pseudoalteromonas* species. (Green, blue and black point out algae, invertebrates, and vertebrates, respectively).

Antimicrobial *Pseudoalteromonas* sp.	Marine Host	Ref.
*P. atlantica*	Aplysina cavernicola,	[87,117]
	Argopecten purpuratus,	
	algae	
*P. citrea*	Apostichopus japonicus,	[118,119,120]
	Crenomytilus grayanus,	
	Patinopecten eyssoensis,	
	Fucus evanescens,	
	sponges	
*P. elyakovii*	Crenomytilus grayanus,	[117,121,122,123]
	Anadara broughtoni,	
	Laminaria japonica,	
	Argopecten purpuratus,	
	sponges	
*P. flavipulchra*	Montipora aequituberculata,	[80,124]
	Scophthalmus maximus	
*P. haloplanktis*	Acanthella purpuratus	[125]
*P. issachenkonii*	Fucus evanescens	[126]
*P. luteoviolacea*	Acanthella cavernosa,	[37,78,127,128]
	Montastrae annularis,	
	Ostrea edulis,	
	Amphiroa anceps,	
	Corallina officinalis,	
	Padina australis,	
	Halopteris scoparia	
*P. maricaloris*	Fascaplysinopsis reticulata,	[129,130]
	Avicennia marina	
*P. peptidolytica*	Montipora sp.	[131]
*P. phenolica*	Unassociated marine macroorganisms	
*P. piscicida*	Amphiprion clarkia,	[90,132,133]
	Hymeniacidon perleve,	
	crustacean	
*P. prydzensis*	Argopecten purpuratus,	[83,117,134]
	Holothuria leucospilota,	
	Crassostrea gigas	
*P. rubra*	Mycale armata,	[16,135]
	Caulerpa peltata	
*P. ruthenica*	Crenomytilus grayanus,	[136,137]
	Pactinopecten yessoensis,	
	Litopenaeus vannamei	
*P. tunicata*	Austrocochlea concamerata,	[32,138,139]
	Ciona intestinalis,	
	Ulva lactuca	
*P. undina*	*Dicentrarchus labrax, Sparus aurata*	[140]
